# From prosocial civic values to online prosocial participation: the roles of self-efficacy, moral responsibility, and public-issue exposure

**DOI:** 10.3389/fpsyg.2026.1887605

**Published:** 2026-09-11

**Authors:** Yue Wang, Ping Zhang

**Affiliations:** 1 School of Marxism, Wuhan Polytechnic University, Wuhan, China; 2 School of Marxism, Hubei University of Chinese Medicine, Wuhan, China

**Keywords:** moral responsibility, online prosocial participation, online prosocial self-efficacy, prosocial civic value orientation, public-issue exposure frequency, structural equation modeling

## Abstract

**Introduction:**

Digital platforms have become important spaces for young people’s public expression, mutual support, and civic engagement, yet less is known about how prosocial civic values are translated into online prosocial participation. This study aimed to examine the psychological mechanisms and contextual activation conditions linking prosocial civic value orientation to online prosocial participation among college students.

**Methods:**

Using questionnaire data from 559 college students in China, this study employed structural equation modeling to test a moderated mediation model, with online prosocial self-efficacy and moral responsibility as mediating variables and Public-issue exposure frequency as a moderating variable.

**Results:**

The results showed that prosocial civic value orientation significantly and positively predicted online prosocial participation. Online prosocial self-efficacy and moral responsibility partially mediated this relationship, while public-issue exposure frequency significantly strengthened the positive association between prosocial civic value orientation and online prosocial participation.

**Discussion:**

These findings suggest that online prosocial participation is shaped by the joint effects of value endorsement, psychological transformation, and digital contextual activation, providing empirical evidence for digital citizenship education in higher education.

## 1 Introduction

The growing integration of digital platforms into everyday life is reshaping how college students engage in public life. For contemporary college students, online spaces are no longer merely instrumental venues for accessing information and maintaining everyday communication. They have increasingly become important arenas for value expression, public discussion, mutual support, and participation in public interest activities. On social media, campus online communities, and other digital platforms, college students engage with public issues through commenting, reposting, information sharing, online mutual assistance, and participation in public interest initiatives, thereby displaying varying levels of online prosocial participation. These behaviors are not only associated with individual digital literacy and the capacity for public participation, but also reflect young people’s value orientations, sense of responsibility, and modes of action in the digital era. Therefore, examining the psychological mechanisms underlying college students’ online prosocial participation has important theoretical and practical significance for understanding how young people enact values in digital public life.

A substantial body of research has examined online prosocial behavior, civic participation, and digital public life, attributing individual differences to factors such as personality traits, Internet use, social support, peer influence, and platform environments (Benvenuti et al., 2024; Gustafsson et al., 2025; Hui et al., 2024; Leng et al., 2020; Pastor et al., 2024). Yet a more fundamental theoretical question remains insufficiently addressed: how do prosocial values endorsed by individuals translate into concrete action in digital public life? College students may endorse public responsibility, mutual aid and cooperation, respect for differences, and collective well-being without necessarily acting to help others, engaging in constructive public expression, or participating in public-interest mutual assistance (Hsu et al., 2021; Jiang et al., 2021). Thus, the central challenge is not merely to identify additional antecedents of online prosocial participation, but to explain how relatively stable value commitments bridge the gap between values and behavior and become translated into concrete digital actions situated within particular contexts. This question is especially salient in digital public life. Although digital platforms reduce barriers to expression and participation, value endorsement does not automatically lead to action. Individuals must still assess whether they can act effectively, whether they feel morally responsible for acting, and whether the immediate context provides sufficient issue-related cues and opportunities for participation (Karliani et al., 2019).

Against this background, the present study conceptualizes online prosocial participation as a process of value realization and examines how prosocial civic value orientation is translated into digital action through proximal psychological mechanisms. Specifically, online prosocial self-efficacy captures an agentic assessment of whether individuals believe they can act effectively, whereas moral responsibility captures a normative assessment of whether they believe they ought to act. Together, these mechanisms represent complementary psychological pathways through which prosocial value commitments are converted into behavioral engagement (House et al., 2020). At the same time, value realization is embedded within the surrounding information environment. Greater Public-Issue Exposure Frequency can increase the visibility of others’ needs and public problems while providing clearer objects of action and more salient cues for participation (Chan et al., 2024). It may therefore shape the extent to which prosocial civic value orientation is translated into online prosocial participation. Accordingly, the central theoretical question of this study is how prosocial values are converted into online participation through agentic and normative psychological mechanisms, and in what digital contexts this conversion is more likely to occur.

Using questionnaire data from college students in Chinese universities, this study employs structural equation modeling to examine the relationships among prosocial civic value orientation, online prosocial self-efficacy, moral responsibility, public-issue exposure frequency, and online prosocial participation. The main contributions of this study are threefold. It extends the explanatory framework of online prosocial participation from the perspective of values, reveals the dual psychological mechanisms through which value orientations are translated into online behavior, and further clarifies the contextual role of exposure to digital public issues in value activation and behavioral enactment. In doing so, this paper deepens the understanding of the formation mechanisms of college students’ online prosocial participation and provides empirical evidence for digital citizenship education and online moral education practices in higher education institutions.

## 2 Literature review and research hypotheses

### 2.1 The value–behavior gap in digital public life

As social media, campus online communities, and various digital platforms have become increasingly integrated into college students’ everyday lives, the arenas in which public participation, mutual support, and value expression take place are no longer confined to offline organizations and local communities, but have extended into online spaces. Online prosocial participation has therefore become an important perspective for understanding young people’s digital public life. It includes behaviors such as providing help and emotional support on digital platforms, sharing useful information, engaging in constructive expression, responding to public issues, and participating in public-interest and mutual-aid activities (Boulianne and Theocharis, 2020). Existing research has defined online prosocial behavior as voluntary behavior that occurs in digital media contexts and is intended to benefit others or promote positive relationships, and has developed corresponding measures for this construct (Erreygers et al., 2018). More recent extensions of these measures have incorporated online emotional support and online action or advocacy into a unified analytical framework, suggesting that online prosocial behavior involves both interpersonal support and public participation (van de Groep et al., 2026).

However, value endorsement does not naturally, directly, or consistently translate into behavioral practice. Research in behavioral science and social psychology has repeatedly shown that even when individuals endorse a particular value in principle, they may fail to act on it because of low perceived efficacy, insufficient contextual cues, a weak sense of responsibility, or high perceived costs of action. This constitutes the core problem of the value-behavior gap (Webb and Sheeran, 2006). The Theory of Planned Behavior offers a broader theoretical insight into this problem: values and attitudinal orientations alone are insufficient to produce behavior directly, because the translation of such orientations into action also depends on proximal psychological assessments and enabling conditions for action (Ajzen, 1991). This issue is especially salient in college students’ digital public life. Online spaces lower the barriers to information dissemination and participatory expression, while also creating obstacles such as fragmented attention, risks associated with public expression, uncertainty in interaction, and bystander mentality (Boulianne and Theocharis, 2020; Weeks et al., 2024). Thus, although prosocial civic value orientation may provide college students with value foundations such as public responsibility, cooperation and mutual aid, respect for differences, and concern for community well-being, whether these values are further translated into online prosocial participation still depends on the presence of relevant psychological resources and contextual cues. Taken together, this paper argues that the stronger college students’ prosocial civic value orientation is, the more likely they are to engage in prosocial participation on digital platforms, including providing help and support, engaging in constructive expression, sharing information, and participating in public-interest and mutual-aid activities. This proposition requires empirical examination. Accordingly, this study proposes the following hypothesis:

*H1:* Prosocial civic value orientation has a significant positive effect on online prosocial participation.

### 2.2 The agentic pathway: online prosocial self-efficacy

Within this framework of value to behavior translation, self-efficacy theory further explains how value endorsement becomes connected to behavioral action through individuals’ judgments of whether they are capable of acting effectively. Self-efficacy theory emphasizes that individuals’ engagement in a given action depends not only on whether they recognize the value of that action, but also on whether they believe they are capable of performing it and achieving the expected outcomes. Bandura emphasized that self-efficacy should be measured with reference to specific domains of action, because individuals’ appraisals of their own capabilities are highly context-specific (Bandura, 1977). In other words, a generalized sense of competence cannot substitute for judgments about one’s capacity to act in specific behavioral contexts. Applying this theoretical logic to digital platforms, online prosocial self-efficacy can be understood as college students’ beliefs about their ability to effectively help others, engage in constructive interaction, and have a positive impact in online spaces (Eastin and LaRose, 2000). Students with stronger prosocial civic value orientation are generally more likely to regard public responsibility, mutual support, and cooperation as meaningful goals for action. They are also more likely to actively seek ways to express goodwill, provide support, and improve the climate of discussion in digital interactions. In this sense, value endorsement does not directly substitute for efficacy beliefs. Rather, it provides a cognitive foundation that helps individuals develop the belief that they can make a difference (Caprara et al., 2012). When college students believe that their comments, reposts, suggestions, comforting messages, or advocacy can have real effects, their willingness to participate is more likely to be translated into online behavior (Hao et al., 2025). Recent research on college students’ online prosocial behavior also shows that online social self-efficacy plays an important psychological bridging role between positive personality traits and online prosocial behavior (Leng et al., 2020), suggesting that efficacy beliefs are an important variable in explaining engagement in digital prosocial action. Accordingly, this paper proposes Hypothesis H2a: Prosocial civic value orientation has a significant positive effect on online prosocial self-efficacy; Hypothesis H2b: Online prosocial self-efficacy has a significant positive effect on online prosocial participation; and further proposes Hypothesis

*H2a:* Prosocial civic value orientation is positively associated with online prosocial self-efficacy.

*H2b:* Online prosocial self-efficacy is positively associated with online prosocial participation.

*H2:* Online prosocial self-efficacy mediates the relationship between prosocial civic value orientation and online prosocial participation.

### 2.3 The normative pathway: moral responsibility

Whereas self-efficacy theory emphasizes whether individuals perceive themselves as capable of acting effectively, the Norm Activation Model further explains why they feel that they ought to act. These two perspectives are complementary rather than substitutive, corresponding, respectively, to capability-based assessments of whether action is feasible and normative judgments of whether action is morally required in the translation of values into behavior. Prosocial behavior is not driven solely by instrumental considerations or emotional impulses. It is also often grounded in individuals’ moral evaluations of the consequences of action, the circumstances and needs of others, and the maintenance of the broader public order. The Norm Activation Model posits that prosocial behavior is closely related to awareness of consequences, ascription of responsibility, and personal norms. When individuals recognize that a given situation may affect others’ well-being and attribute part of the responsibility to themselves, their internal moral norms may be activated, thereby motivating prosocial action (De Groot and Steg, 2009). Existing studies have also shown that awareness, responsibility, and personal norms are key psychological processes in explaining prosocial behavior (Steg and De Groot, 2010).

On digital platforms, individual behaviors often take relatively low-threshold forms, such as commenting, liking, reposting, sending private messages, and sharing information, but their social consequences can still be substantial. A constructive response may provide emotional support to others, sharing reliable information may reduce misunderstandings around public issues, and a rational contribution to discussion may improve the deliberative climate. Conversely, silence, indifference, malicious comments, or the spread of unverified information may likewise affect others’ well-being and the online public environment (Anderson et al., 2014; Oh et al., 2014). Accordingly, college students with a stronger prosocial civic value orientation are more likely to recognize the social consequences of online behavior and to view themselves as responsible agents in digital public spaces. Their online prosocial participation is therefore not merely an expression of willingness to help, but more closely resembles an internalized form of public ethical practice (Choi, 2016). Research on adolescents’ online morality also suggests that moral identity can promote online empathy, authenticity, and moral action, whereas moral disengagement may weaken positive online behavior (Morgan and Fowers, 2021). Based on this reasoning, this paper proposes Hypothesis

*H3a:* Prosocial civic value orientation has a significant positive effect on moral responsibility.

*H3b:* Moral responsibility has a significant positive effect on online prosocial participation.

*H3:* Moral responsibility mediates the relationship between prosocial civic value orientation and online prosocial participation.

### 2.4 Public-issue exposure as a contextual activation condition

The two psychological pathways outlined above explain how value endorsement generates internal motivational forces for action. Yet this process remains embedded in the digital information environment in which individuals encounter issues, needs, and opportunities to participate. Among the various contextual factors within digital environments, this study focuses on public issue exposure frequency because it is directly aligned with the central question of how values are translated into behavior. Compared with broader factors such as general Internet use intensity, social support, or platform climate, exposure to public issues more directly increases the visibility of societal problems and others’ needs while making concrete objects of action and opportunities for participation more salient. Prior research has likewise found that exposure to public affairs information on social media is closely associated with civic and political participation (Gil de Zúñiga et al., 2012; Kim, 2023).

For college students, more frequent exposure to public issues means that they are more likely to encounter social issues, public-interest initiatives, campus public affairs, mutual-aid information, and contexts for public discussion on digital platforms. Such content does more than provide information. It can activate relevant values, evoke emotional resonance, heighten the salience of normative considerations, and supply cues for action, thereby making abstract values more readily applicable to concrete behavioral contexts (Hertz and Krettenauer, 2016). When individuals with a strong prosocial civic value orientation are repeatedly exposed to public issue content, values such as public responsibility, mutual aid and cooperation, and collective well-being are more likely to become salient within specific situations and to be translated into concrete action (Theocharis et al., 2023; Valenzuela et al., 2012). In other words, public-issue exposure frequency can strengthen the predictive effect of value orientation on online prosocial participation, making it easier for individuals to translate internalized values into concrete acts of helping, expression, information sharing, and public-interest participation. Accordingly, this paper proposes Hypothesis

*H4:* Public-issue exposure frequency positively moderates the relationship between prosocial civic value orientation and online prosocial participation.

### 2.5 The integrated moderated mediation model

**Figure 1:**
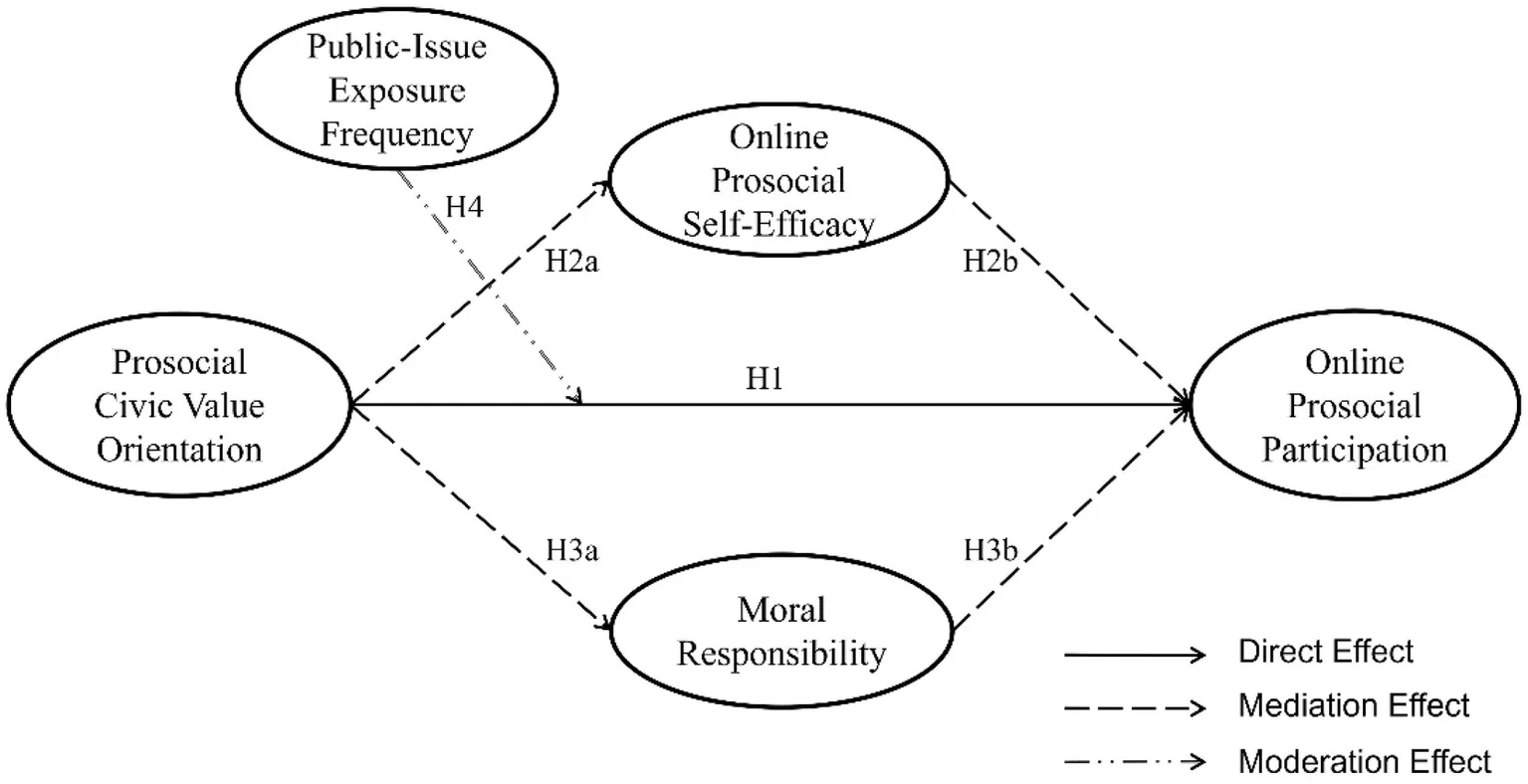
Theoretical model of online prosocial participation among college students.

Based on the foregoing analysis, this study adopts the translation of values into behavior in digital public life as its overarching framework. The Theory of Planned Behavior provides a general account of why value orientations do not automatically translate into action. Self-efficacy theory and the Norm Activation Model further identify two complementary mechanisms, namely an agentic mechanism concerning whether individuals perceive themselves as capable of acting effectively and a normative mechanism concerning whether they feel morally obligated to act. Together, these mechanisms explain the psychological process through which prosocial civic value orientation is translated into online prosocial participation. Furthermore, this study conceptualizes public issue exposure frequency as a contextual condition that activates value realization and examines how the digital information environment shapes the translation of values into behavior. These theoretical elements are integrated into a framework comprising a distal value foundation, dual psychological mechanisms, contextual activation, and behavioral participation. The theoretical model is shown in Figure 1.

## 3 Research design

### 3.1 Data sources

This study surveyed college students enrolled in Chinese universities. To improve the balance of the sample structure and the external validity of the findings, the survey used stratified random sampling while taking into account institutional type, disciplinary background, year of study, and gender composition. Questionnaires were distributed through a combination of online platform-based recruitment and supplementary offline collection in classrooms and campus settings. This approach was intended to broaden survey coverage and minimize potential sample selection bias associated with reliance on a single recruitment channel. Data were collected from late December 2025 to March 2026, yielding 600 returned questionnaires. During data cleaning, invalid responses were excluded using a consistent set of quality control criteria. Specifically, a questionnaire was removed if it met any of the following conditions: more than 10% of the core items were missing; response scores differed by 3 points or more across at least two sets of semantically related items; key background information contained clear logical inconsistencies; completion time was less than one third of the median completion time or more than three times that median; or the response pattern showed clear regularity, defined as selecting the same response option for at least 10 consecutive core items or using a single response option for 80% or more of all core items. Questionnaires meeting any one of these exclusion criteria were removed. The final analytic sample comprised 559 valid questionnaires, corresponding to a validity rate of 93.2%. With respect to the adequacy of the sample size relative to model complexity, the present study specified 19 observed variables. According to a commonly applied rule of thumb in structural equation modeling, the sample size should generally be approximately 5 to 10 times the number of observed variables (Kendall, 1957). The sample size in this study exceeds the minimum empirical requirement by a substantial margin, and is therefore sufficient to support subsequent SEM estimation and robustness tests.

### 3.2 Variable selection and descriptive statistics

The variables in this study were specified according to a theoretical chain linking value orientation, psychological mechanisms, contextual conditions, and behavioral outcomes. Drawing on established scales and relevant empirical studies, the measurement items required for the model were adapted to the context in which Chinese college students participate on digital platforms (see Table 1). Most questionnaire items were measured on a five-point Likert scale. Items measuring attitudinal endorsement used response options ranging from 1, “strongly disagree,” to 5, “strongly agree,” to assess respondents’ endorsement of values, efficacy beliefs, and sense of responsibility. Frequency-based indicators, including public-issue exposure frequency and online prosocial participation, used five ordered response categories ranging from “never” to “very frequently,” in order to capture individual differences in exposure and behavioral engagement on digital platforms. To ensure the content validity and response consistency of the measurement instrument, questionnaire items were organized into modules according to their latent constructs. Item development followed a sequential procedure comprising literature review, identification and refinement of construct dimensions, contextual adaptation, small sample pilot testing, and subsequent adjustments to wording and item order. Prior to the formal survey, the questionnaire was pilot tested with 20 college students to assess the clarity of item wording, ease of semantic comprehension, and potential sources of ambiguity. Based on participant feedback, the wording of selected items and the sequence of certain items were refined without altering the construct definitions or core measurement content. This process was intended to maintain close logical alignment between the measurement content and the theoretical framework while ensuring the feasibility of empirical implementation. The complete items and response formats are provided in Appendix A.

**Table 1:** Measurement items for latent variables.

Variable category	Latent variable	Item	Observed variable	References
Independent variable	Prosocial civic value orientation (PCV)	PCV1	Public responsibility endorsement	Doolittle and Faul (2013) and Park and Hong (2025)
		PCV2	Mutual support and cooperation orientation	
		PCV3	Respect for and inclusion of differences	
		PCV4	Community well-being orientation	
Mediating variables	Online prosocial self-efficacy (OPS)	OPS1	Perceived ability to help others online	Feng et al. (2025) and Leng et al. (2020)
		OPS2	Perceived ability to engage in constructive interaction	
		OPS3	Perceived ability to exert a positive influence	
	Moral responsibility (MR)	MR1	Awareness of behavioral consequences	De Groot and Steg (2009) and Savari et al. (2023)
		MR2	Responsibility for others’ well-being	
		MR3	Obligation to participate constructively	
		MR4	Self-restraint and taking responsibility	
Moderating variable	Public-issue exposure frequency (PIE)	PIE1	Frequency of exposure to social issues	Dreston and Neubaum (2023) and Gil de Zúñiga et al. (2017)
		PIE2	Frequency of exposure to public-interest and mutual-aid issues	
		PIE3	Frequency of exposure to campus public affairs	
		PIE4	Frequency of exposure to public discussions and comments	
Dependent variable	Online prosocial participation (OPP)	OPP1	Online help and support	Erreygers et al. (2018), Nuttall et al. (2025), and van de Groep et al. (2026)
		OPP2	Constructive expression	
		OPP3	Useful information sharing	
		OPP4	Online mutual-aid and public-interest participation	

After completing the scale design and item organization, this study calculated descriptive statistics for the main survey sample to describe the composition of the respondents and provide basic information for subsequent model estimation (see Table 2). A total of 559 valid responses were obtained. Among the respondents, 258 were male, accounting for 46.2%, and 301 were female, accounting for 53.8%, indicating a generally balanced gender distribution. By year of study, 132 respondents were first-year students, accounting for 23.6%; 155 were second-year students, accounting for 27.7%; 173 were third-year students, accounting for 30.9%; and 99 were fourth-year students, accounting for 17.7%. The sample therefore covered different stages of undergraduate study. In terms of urban or rural background, 380 respondents came from urban areas, accounting for 68.0%, whereas 179 came from rural areas, accounting for 32.0%. Regarding family structure, 468 respondents were only children, accounting for 83.7%, whereas 91 had siblings, accounting for 16.3%. Overall, the sample shows variation in gender, year of study, urban or rural background, and family structure, providing a reasonably robust empirical basis for subsequent measurement model testing and structural path analysis. In addition, correlation analysis and variance inflation factor tests were conducted for the main variables. The results showed no evidence of significant multicollinearity, indicating that the data met the basic assumptions required for structural equation modeling.

**Table 2:** Descriptive statistics of samples.

Variable	Variable groups	Frequency	Percentage
Genders	Male	258	46.2%
	Female	301	53.8%
Grade	First grade	132	23.6%
	Second grade	155	27.7%
	Third grade	173	30.9%
	Fourth grade	99	17.7%
Urban or rural	Urban	380	68.0%
	Rural	179	32.0%
Single child or not	Yes	468	83.7%
	No	91	16.3%

### 3.3 Research methods

To systematically examine the mechanisms and contextual activation through which college students’ prosocial civic value orientation influences online prosocial participation, this study employed structural equation modeling for empirical analysis. Preliminary assessments of data quality and common method bias were first conducted to confirm that the sample data met the basic requirements for model estimation. Following the two-step approach commonly used in structural equation modeling, the study first used confirmatory factor analysis to examine the relationships between latent constructs and observed indicators, and then estimated the structural model to test the direct effect of prosocial civic value orientation on online prosocial participation, as well as the mediating effects of online prosocial self-efficacy and moral responsibility. The mediating effects were tested using the bias-corrected bootstrap resampling method to obtain more robust confidence intervals for the indirect effects. Meanwhile, an interaction term was introduced to examine whether public-issue exposure frequency moderated the path from prosocial civic value orientation to online prosocial participation. Simple slope analysis was then used to further illustrate how the focal relationship varied across different levels of public-issue exposure. These analyses were mainly conducted using SPSS 27.0 and AMOS 28.0, allowing the analytical procedure to proceed systematically from measurement model testing to structural path estimation and the identification of mediation and moderation mechanisms.

## 4 Empirical results analysis

### 4.1 Model fit test

#### 4.1.1 Test for common method variance

Before estimating the structural equation model, this study first examined whether the questionnaire data were affected by common method variance. Because the core variables were measured using the same questionnaire survey, Harman’s single-factor test and a CFA-based marker-variable approach were used as complementary checks. The results showed that, in the unrotated factor analysis, the first principal component explained less than the conventional 50% threshold of the total variance, indicating that no single factor dominated the variance in the data (Harman, 1986). The results of the CFA-based marker-variable test also provided no evidence of serious common method effects (Lindell and Whitney, 2001). Therefore, the data used in this study did not exhibit significant common method bias, and common method variance is unlikely to materially affect subsequent model estimation.

#### 4.1.2 Reliability, validity, and confirmatory factor analysis

To examine the reliability and validity of the measurement structure, this study further conducted reliability and validity tests as well as confirmatory factor analysis. The results are reported in Table 3. Overall, the standardized factor loadings of the observed variables ranged from 0.719 to 0.958, all reaching acceptable levels. Except for the fixed reference indicators, the unstandardized loadings of all remaining items were significant at the 1% level, indicating that the observed indicators effectively reflected their corresponding latent variables. The composite reliability (CR) values of the latent variables ranged from 0.932 to 0.960, all exceeding the conventional threshold of 0.70, which indicates good internal consistency of the scales (Hair et al., 1998). The average variance extracted (AVE) values ranged from 0.785 to 0.858, all exceeding the threshold of 0.50, indicating satisfactory convergent validity for each latent variable (Kline, 1998). These results suggest that the measurement model is reliable overall and provides an adequate basis for subsequent structural path analysis.

**Table 3:** Results of reliability and validity and confirmatory factor analysis.

Latent variable	Item	Parameter significance estimation				Factor loading (Std.)	CR	AVE
		Unstd.	S.E.	t-value	*p*-value			
Prosocial civic value orientation (PCV)	PCV1	1				0.922	0.960	0.857
	PCV2	0.861	0.026	32.65	***	0.869		
	PCV3	0.934	0.022	43.135	***	0.952		
	PCV4	0.925	0.021	44.008	***	0.957		
Online prosocial self-efficacy (OPS)	OPS1	1				0.891	0.932	0.822
	OPS2	0.914	0.03	30.23	***	0.879		
	OPS3	0.957	0.027	34.989	***	0.948		
Moral responsibility (MR)	MR1	1				0.901	0.960	0.858
	MR2	0.927	0.027	34.005	***	0.903		
	MR3	0.885	0.023	39.133	***	0.949		
	MR4	0.912	0.023	39.366	***	0.951		
Public-issue exposure frequency (PIE)	PIE1	1				0.935	0.944	0.808
	PIE2	0.673	0.025	26.968	***	0.797		
	PIE3	0.894	0.023	39.684	***	0.923		
	PIE4	0.920	0.022	41.171	***	0.934		
Online prosocial participation (OPP)	OPP1	1				0.719	0.935	0.785
	OPP2	1.206	0.056	21.373	***	0.900		
	OPP3	1.199	0.053	22.744	***	0.958		
	OPP4	1.15	0.051	22.472	***	0.946		

*, **, *** indicate significant at the statistical level of 10, 5, and 1%, respectively.

To examine discriminant validity, this study applied the Fornell-Larcker criterion by comparing the square root of the AVE for each latent variable with the correlations among latent variables. As shown in Table 4, the square root of the AVE for each latent variable was higher than its correlations with all other latent variables. In addition, the highest correlation coefficient among the latent variables was 0.373, indicating no substantial overlap among the constructs. These results suggest that the latent variables have clear empirical boundaries and that the scales demonstrate good discriminant validity.

**Table 4:** Discriminant validity test results.

Latent variable	The square root of the AVE value	PCV	OPS	MR	PIE	OPP
PCV	0.857	**0.926**				
OPS	0.822	0.293	**0.907**			
MR	0.858	0.353	0.373	**0.926**		
PIE	0.808	0.132	0.157	0.130	**0.899**	
OPP	0.785	0.231	0.228	0.244	0.139	**0.886**

Bold values on the diagonal represent the square roots of the average variance extracted (AVE) for each latent variable.

#### 4.1.3 Model fit assessment

Before estimating the structural paths, this study further assessed the overall fit between the proposed model and the sample data. Model fit was evaluated using three categories of indices, namely absolute fit indices, incremental fit indices, and parsimonious fit indices, to avoid biased conclusions resulting from reliance on a single index. The AMOS 28.0 estimation results showed that the main fit indices of the modified model met acceptable thresholds (see Table 5). Specifically, the absolute fit indices, including χ^2^/df, SRMR, and GFI, the incremental fit indices, including IFI, NFI, and CFI, and the parsimonious fit indices, including PCFI and PNFI, all satisfied the commonly accepted criteria in structural equation modeling research. These results indicate that the modified structural model showed a good fit to the sample data and was suitable for subsequent tests of path effects, mediating effects, and moderating effects.

**Table 5:** Results of the model fit assessment.

Fit indices	Absolute adaptation index			Value-added adaptation indicators			Simple adaptation index	
	χ^2^/df	RMSEA	GFI	IFI	NFI	CFI	PCFI	PNFI
Adaptation criteria	<3	<0.08	>0.90	>0.90	>0.90	>0.90	>0.50	>0.50
Test results	2.963	0.059	0.910	0.972	0.958	0.972	0.826	0.814
Adaptation results	Yes	Yes	Yes	Yes	Yes	Yes	Yes	Yes

### 4.2 Hypothesis testing

#### 4.2.1 Analysis of direct effects

Table 6 presents the path analysis results for the structural model. Overall, all hypothesized paths in the model were statistically significant at the 1% level, indicating that the proposed analytical framework linking prosocial civic value orientation, psychological mechanisms, and online prosocial participation was empirically supported. Specifically, prosocial civic value orientation had a significant positive effect on online prosocial participation (β = 0.203, t = 8.871, *p* < 0.001). This result indicates that the stronger college students’ endorsement of values such as public responsibility, mutual support and cooperation, and community well-being, the more likely they are to engage in prosocial behaviors on digital platforms, including helping others, constructive expression, information sharing, and participation in public-interest and mutual-aid activities. Thus, Hypothesis H1 was supported. Online prosocial self-efficacy also significantly and positively predicted online prosocial participation (β = 0.288, t = 11.734, *p* < 0.001), suggesting that individuals’ beliefs in their ability to help others online, engage in constructive interaction, and exert a positive influence constitute an important psychological driver that helps translate value endorsement into actual online prosocial behavior. Thus, Hypothesis H2b was supported. Moral responsibility likewise significantly promoted online prosocial participation (β = 0.274, t = 12.698, *p* < 0.001), indicating that the stronger college students’ sense of responsibility for the consequences of online behavior, others’ well-being, and the norms of public interaction, the more likely they are to adopt constructive and altruistic forms of participation in digital spaces. Thus, Hypothesis H3b was supported.

**Table 6:** Path analysis results for the structural model.

Path	Estimate	S.E.	t-value	*p*	Result
H1: Prosocial civic value orientation → Online prosocial participation	0.203^***^	0.023	8.871	<0.001	Supported
H2b: Online prosocial self-efficacy → Online prosocial participation	0.288^***^	0.025	11.734	<0.001	Supported
H3b: Moral responsibility → Online prosocial participation	0.274^***^	0.022	12.698	<0.001	Supported
H2a: Prosocial civic value orientation → Online prosocial self-efficacy	0.500^***^	0.039	12.943	<0.001	Supported
H3a: Prosocial civic value orientation → Moral responsibility	0.577^***^	0.043	13.481	<0.001	Supported
H4: PCV × PIE → Online prosocial participation	0.050^**^	0.021	2.400	0.016	Supported

*, **, *** indicate significant at the statistical level of 10, 5, and 1%, respectively.

Meanwhile, prosocial civic value orientation also significantly and positively predicted online prosocial self-efficacy (β = 0.500, t = 12.943, *p* < 0.001) and moral responsibility (β = 0.577, t = 13.481, *p* < 0.001). This finding indicates that value orientation is not limited to abstract normative endorsement, but can be further translated into individuals’ efficacy beliefs and sense of responsibility, thereby providing a psychological foundation for subsequent behavioral engagement. Accordingly, Hypotheses H2a and H3a were also supported. Taken together, these findings suggest that prosocial civic value orientation not only directly promotes online prosocial participation, but also provides sustained cognitive and normative support for prosocial action in the digital public spaces in which college students participate by shaping their efficacy beliefs and sense of responsibility.

#### 4.2.2 Mediation analysis

To further examine the psychological mechanisms linking prosocial civic value orientation to online prosocial participation, this study conducted bootstrap mediation analysis with online prosocial self-efficacy and moral responsibility as mediators. As shown in Table 7, the total effects for both hypothesized mediation paths were significant. The indirect effect through online prosocial self-efficacy was significant (β = 0.074, S.E. = 0.017, t = 4.353, 95% CI = [0.044, 0.111]), supporting Hypothesis H2. The indirect effect through moral responsibility was also significant (β = 0.077, S.E. = 0.016, t = 4.813, 95% CI = [0.050, 0.110]), supporting Hypothesis H3. Because the confidence intervals for both indirect effects excluded zero, the results indicate that both online prosocial self-efficacy and moral responsibility served as significant mediating mechanisms. In addition, the direct effect of prosocial civic value orientation on online prosocial participation remained significant after the mediators were included (β = 0.188, S.E. = 0.020, t = 9.400, 95% CI = [0.150, 0.231]), suggesting partial mediation. Overall, these findings indicate that prosocial civic value orientation is associated with online prosocial participation not only directly, but also indirectly through students’ efficacy beliefs and moral responsibility.

**Table 7:** Results of mediation effect test.

Mediation path	Estimate	S.E.	t-value	95% Confidence Interval	
				Lower	Upper
Total effects					
*H2:* Prosocial civic value orientation → Online prosocial self-efficacy → Online prosocial participation	0.347^***^	0.030	11.57	0.286	0.403
*H3:* Prosocial civic value orientation → Moral responsibility → Online prosocial participation	0.361^***^	0.028	12.89	0.308	0.419
Indirect effects					
*H2:* Prosocial civic value orientation → Online prosocial self-efficacy → Online prosocial participation	0.074^***^	0.017	4.353	0.044	0.111
*H3:* Prosocial civic value orientation → Moral responsibility → Online prosocial participation	0.077^***^	0.016	4.813	0.050	0.110
Direct effects					
*H1:* Prosocial civic value orientation → Online prosocial participation	0.188^***^	0.020	9.400	0.150	0.231

*, **, *** indicate significant at the statistical level of 10, 5, and 1%, respectively.

#### 4.2.3 Moderation analysis

**Figure 2:**
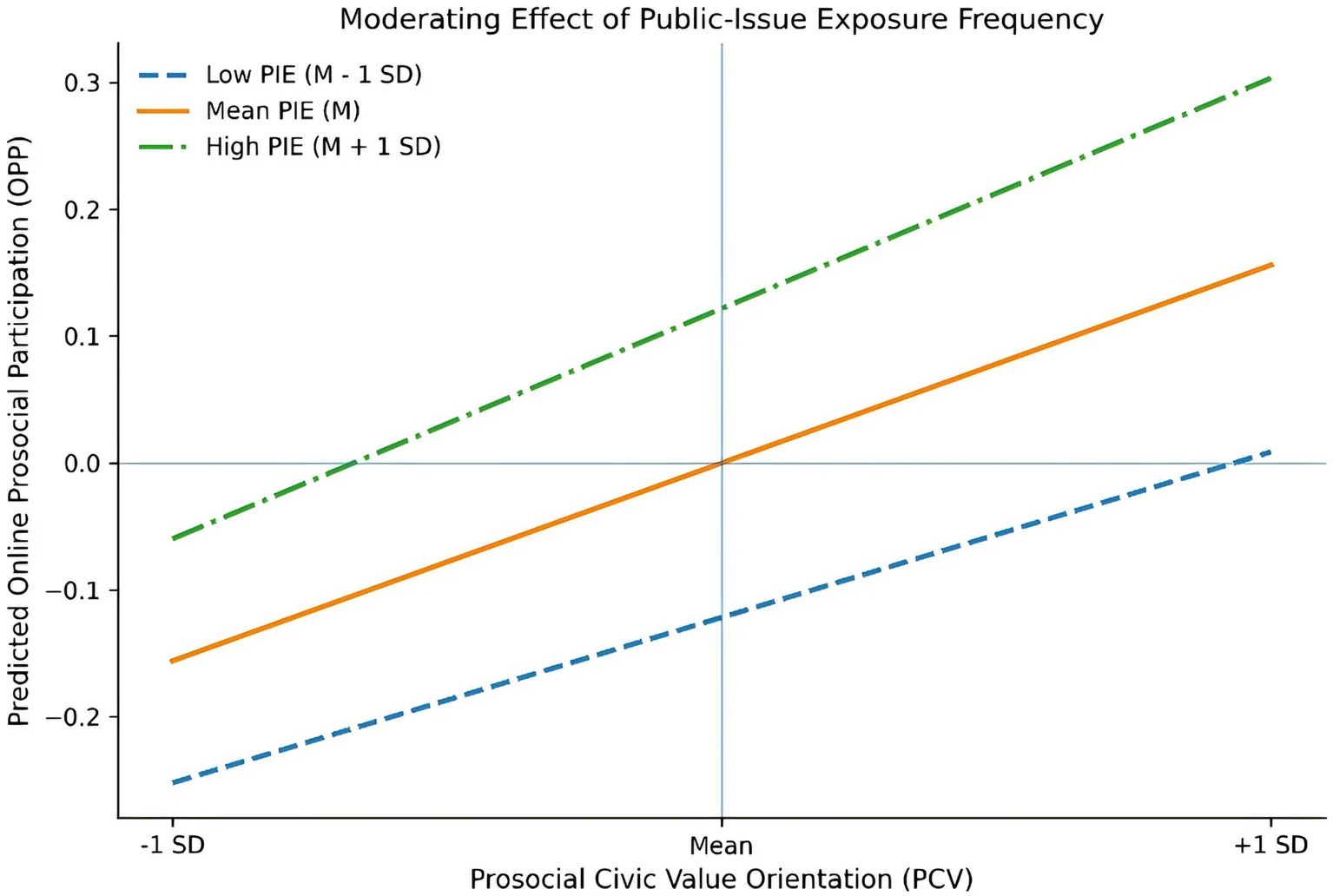
Moderating effect of public-issue exposure frequency.

To examine the moderating role of public-issue exposure frequency in the relationship between prosocial civic value orientation and online prosocial participation, this study included an interaction term in the structural model. The results showed that the interaction term had a significant positive effect on online prosocial participation (β = 0.050, t = 2.400, *p* < 0.05), indicating that public-issue exposure frequency significantly strengthened the positive relationship between prosocial civic value orientation and online prosocial participation. Thus, Hypothesis H4 was supported. Further inspection of the moderation plot in Figure 2 shows that, at high levels of public-issue exposure, the positive slope linking prosocial civic value orientation and online prosocial participation was more pronounced, whereas at low levels of public-issue exposure, this relationship was relatively weaker. These findings suggest that public-issue exposure frequency not only reflects the extent to which college students are exposed to public-issue information, but also serves as an important contextual condition under which value endorsement is translated into online prosocial action. More frequent exposure to public issues can provide individuals with more cues for action, more contexts for public expression, and more opportunities for social connection, thereby strengthening the extent to which prosocial civic value orientation is translated into behavior. Overall, public-issue exposure frequency constitutes a key contextual condition in the transformation of value orientation into behavioral enactment, further revealing the heterogeneous nature of the mechanisms underlying college students’ prosocial participation within digital platform environments.

To further assess whether public issue exposure frequency moderated the indirect effects, this study employed Hayes’s PROCESS procedure to test moderated mediation (Hayes, 2015). As shown in Table 8, for the “PCV → OPS → OPP” pathway, the conditional indirect effects were significant at low, medium, and high levels of PIE, with all 95% bootstrap confidence intervals excluding zero. However, the index of moderated mediation was −0.020, with a 95% bootstrap CI of [−0.054, 0.015]. Because this interval included zero, there was no significant evidence of moderated mediation for this pathway. Similarly, for the “PCV → MR → OPP” pathway, the conditional indirect effects were significant at low, medium, and high levels of PIE, with all 95% bootstrap confidence intervals excluding zero. Nevertheless, the index of moderated mediation was 0.032, with a 95% bootstrap CI of [−0.008, 0.072]. As this interval also included zero, the moderated mediation effect for this pathway was not significant. Overall, PIE did not significantly alter the magnitude of either indirect effect.

**Table 8:** Bootstrap test of moderated mediation.

Indirect path	Level of PIE	Estimate	Boot SE	Z	*p*	Boot LLCI	Boot ULCI
PCV → OPS → OPP	Low	0.128	0.019	6.88	<0.001	0.093	0.166
	Median	0.113	0.016	7.04	<0.001	0.084	0.147
	High	0.109	0.018	6.16	<0.001	0.075	0.145
	Index of moderated mediation	−0.020	0.018	−1.10	0.273	−0.054	0.015
PCV → MR → OPP	Low	0.098	0.019	5.21	<0.001	0.061	0.137
	Median	0.122	0.017	7.11	<0.001	0.090	0.157
	High	0.130	0.019	6.68	<0.001	0.094	0.170
	Index of moderated mediation	0.032	0.020	1.56	0.119	−0.008	0.072

## 5 Discussion

Focusing on the mechanisms underlying college students’ prosocial participation on digital platforms, this study constructed and tested a structural equation model in which prosocial civic value orientation served as the starting point, online prosocial self-efficacy and moral responsibility served as psychological pathways, and public-issue exposure frequency served as a contextual boundary condition. The findings indicate that online prosocial participation is not a direct behavioral expression of value endorsement. Rather, it emerges through a process in which value orientations are translated into action through judgments of one’s capacity to act and the internalization of moral responsibility, while also being shaped by specific digital contexts. College students’ online prosocial behavior therefore cannot be reduced to immediate emotional reactions or habitual patterns of platform use; instead, it reflects the joint operation of value endorsement, psychological processes, and the surrounding digital context. By shifting attention from the question of “what factors predict online prosocial participation” to the question of “how prosocial values are psychologically translated into digital action,” this study provides a more explicitly process based account of how the value to behavior gap emerges and may be bridged in digital public life.

First, the significant positive association between prosocial civic value orientation and online prosocial participation suggests that college students who more strongly endorse values of public responsibility, mutual aid and cooperation, respect for differences, and collective well-being are more likely to engage in constructive action in digital spaces. Prior research on civic engagement has long suggested that civic attitudes, social responsibility, awareness of social justice, and respect for differences constitute important psychological foundations for youth public participation (Moely et al., 2002). Measures such as the CASQ and the Civic Engagement Scale likewise incorporate civic responsibility, social justice, attitudes toward diversity, and behavioral orientations as central dimensions of youth civic development (Doolittle and Faul, 2013), while digital participation has become an important component of contemporary civic engagement (Park and Hong, 2025). More importantly, the present findings suggest that the role of value orientation cannot be reduced to a simple pattern in which stronger values correspond to greater participation. Its significance may instead lie in shaping how individuals interpret situations encountered online. College students with a stronger prosocial civic value orientation may be more likely to perceive others’ needs, public problems, and conflicts arising in discussion as situations that warrant a response, thereby construing helping, sharing, and constructive expression as meaningful forms of action. Prosocial civic value orientation may therefore function not merely as an abstract form of normative endorsement but as a psychological foundation for recognizing public needs, evaluating the significance of possible actions, and selecting appropriate modes of participation. From this perspective, digital platforms are more than technological environments in which behavior takes place; they may also constitute important public spaces through which individuals enact and express the values they endorse.

Second, the mediating role of online prosocial self-efficacy indicates that the translation of value endorsement into behavioral practice requires the efficacy belief that one is able to act. Bandura’s classic account of self-efficacy emphasizes that self-efficacy should be understood as individuals’ judgments about whether they can perform specific tasks, rather than as a generalized positive self-evaluation (Bandura, 1977, 2006). In the context of digital platforms, even when college students endorse prosocial values, civic responsibility, and public well-being, their value orientation may remain at the attitudinal level if they lack confidence in their ability to help others effectively, communicate rationally, or exert a positive influence in online environments. In such cases, it may be difficult for these values to translate into sustained online participation. From a psychological process perspective, self-efficacy may function as an “action feasibility filter.” Prosocial values may first render certain actions meaningful and worth pursuing, but these values are more likely to shape actual behavioral choices only when individuals believe that they can express themselves effectively, support others, or make a positive difference. Prosocial civic value orientation may therefore foster more proactive prosocial behavior in online spaces by strengthening individuals’ confidence in their capacity to act effectively in digital contexts. This finding extends the explanatory relevance of self-efficacy theory to digital prosocial behavior and further suggests that, for values education in universities to translate into online public action, it cannot focus solely on why students “ought to participate.” It must also strengthen students’ perceived capacity to “participate effectively.”

Third, the mediating effect of moral responsibility further reveals the mechanism of normative internalization through which value endorsement is translated into online prosocial participation. The norm activation model suggests that prosocial behavior is often based on the activation of awareness of consequences, ascription of responsibility, and personal norms. When individuals recognize that their behavior may affect others’ well-being and link those consequences to their own responsibility, prosocial action is more likely to be understood as an internal moral obligation (De Groot and Steg, 2009; Savari et al., 2023). From this perspective, prosocial civic value orientation may strengthen individuals’ awareness of the consequences of online behavior and their sense of personal responsibility, thereby converting abstract value commitments into more compelling subjective obligations. On digital platforms, prosocial behavior is therefore not merely a matter of choosing to act when one feels capable of doing so; it is also shaped by a normative sense that one is responsible for acting. This mechanism may be particularly salient in online spaces, where posting, sharing, commenting, and even passive observation can have broader consequences for others’ emotions, the climate of public discussion, and the information ecology. Moral Responsibility may therefore serve as an important psychological link between value orientation and behavioral enactment (Brady et al., 2017; Steg and De Groot, 2010). Notably, the two indirect effects were similar in magnitude, suggesting that the agentic judgment that one is capable of acting effectively and the normative judgment that one ought to act each provide distinct explanatory value. Online prosocial participation should therefore not be understood as the product of a single motivational force but as more likely to emerge from the combined support of perceived capacity for action and a sense of moral obligation.

Furthermore, the moderating role of public issue exposure frequency suggests that the translation of value orientation into online prosocial participation varies across digital contexts and is meaningfully shaped by the surrounding information environment. The psychological significance of such exposure may lie less in simply increasing the volume of information encountered than in making public issues, others’ needs, and opportunities for action more visible, thereby linking abstract values to concrete targets and immediately available cues for participation. Exposure to public issues therefore does more than provide additional information; more importantly, it supplies contextual cues that can activate relevant values. When college students with a stronger prosocial civic value orientation repeatedly encounter public issue content, their existing value commitments may be more readily activated by concrete problems, visible needs, and situations of public discussion, increasing the likelihood that these commitments are enacted through online behaviors such as commenting, sharing, expressing support, advocating, and providing mutual aid. Public issue exposure frequency may therefore operate as a form of “contextual activation mechanism” by bringing abstract values into closer contact with concrete opportunities for action and, in turn, increasing the likelihood that values are enacted in practice. The moderated mediation results further refine this interpretation. Although public issue exposure frequency strengthened the conditional direct association between prosocial civic value orientation and online prosocial participation, it did not significantly alter the indirect effects operating through online prosocial self-efficacy or moral responsibility. This pattern suggests that the influence of digital context may be pathway specific. Exposure to public issues appears primarily to facilitate the more immediate behavioral expression of values by increasing the visibility of relevant problems and opportunities for action, whereas the psychological mechanisms concerning whether individuals feel capable of acting effectively and morally obligated to act remain comparatively consistent across levels of exposure. Online prosocial participation may therefore be better understood as emerging from the joint operation of relatively stable psychological mechanisms and dynamically activated contextual conditions, rather than from a general amplification of all psychological processes through greater information exposure.

Taken as a whole, this study makes three main theoretical contributions. First, it incorporates prosocial civic value orientation into the explanatory framework of college students’ online prosocial participation, thereby extending prior research that has largely accounted for online prosocial behavior in terms of personality traits, emotional states, and patterns of media use. In doing so, the study highlights the foundational role of value orientations in shaping digital prosocial engagement. Second, it distinguishes two complementary psychological mechanisms through which values are translated into behavior. Online prosocial self-efficacy captures an agentic assessment of whether individuals perceive themselves as capable of acting effectively, whereas moral responsibility captures a normative assessment of whether they feel obligated to act. By explaining value enactment from the distinct perspectives of agency and normativity, these mechanisms provide a more differentiated account of how value endorsement is translated into digital prosocial behavior. Third, the study incorporates public issue exposure frequency into the model, demonstrating that the digital information environment is not merely a passive backdrop but an important contextual condition for the enactment of values. This contribution further connects individual psychological processes with the surrounding digital issue environment, suggesting that online prosocial participation depends both on internal psychological mechanisms and on the contextual activation generated by concrete issue cues and opportunities for action.

This study also has practical implications. If values education in universities is to effectively promote college students’ prosocial participation in digital spaces, it should not remain limited to the transmission of abstract ideas or normative advocacy. Instead, it should also focus on capacity building, responsibility cultivation, and the creation of enabling contexts. On the one hand, universities can strengthen students’ abilities to express themselves rationally, help others effectively, and interact constructively in online environments through digital citizenship education, digital literacy courses, public-issue discussions, online volunteer service, and campus public-interest communication projects. These efforts can provide students with clearer pathways for putting prosocial values into practice. On the other hand, universities should strengthen students’ sense of responsibility for the consequences of online behavior, others’ well-being, and the norms of public interaction, guiding them to view digital spaces as social spaces with ethical constraints and public significance. In addition, platforms and universities can provide students with more high-quality, accessible, and sustainable opportunities for online prosocial participation by optimizing the supply of public-issue content, expanding access points for public-interest participation, and fostering a climate of rational discussion. These efforts can increase the likelihood that value endorsement will be translated into behavioral practice.

Nevertheless, several limitations should be acknowledged. First, the use of cross-sectional survey data precludes firm conclusions regarding the temporal ordering and causal direction of the relationships among the study variables. Accordingly, the findings concerning direct associations, indirect pathways, and moderation should be interpreted as theory informed associational evidence rather than as strict causal identification. Future research could employ multiwave longitudinal designs, cross lagged models, and experimental or quasi experimental approaches to examine potential reverse relationships and more rigorously assess the proposed causal mechanisms. Second, the core constructs were measured primarily through self-reports. Although the diagnostic tests conducted in this study did not indicate substantial common method bias, potential influences arising from common source measurement, socially desirable responding, and consistency motives cannot be entirely ruled out. Future studies could integrate self-report measures with behavioral trace data from digital platforms, peer assessments, or indicators derived from experimental tasks to enhance the objectivity of behavioral measurement. Third, the present sample was restricted to college students in China. Whether the findings generalize across regions, institutional types, and cross-cultural contexts remains to be examined using broader and more diverse samples. Finally, public-issue exposure frequency mainly captured the level of exposure, but did not fully distinguish more fine-grained digital contextual factors, such as issue type, information quality, algorithmic recommendation mechanisms, and interaction climate. Future research could further expand the dimensions of contextual variables to provide a deeper understanding of how digital platform environments shape the activation, translation, and enactment of college students’ prosocial values.

## 6 Conclusion

Based on survey data from college students in Chinese universities, this study constructed and tested a structural equation model linking prosocial civic value orientation to online prosocial participation. The results showed that prosocial civic value orientation significantly enhanced college students’ online prosocial participation, indicating that endorsement of values such as public responsibility, mutual support and cooperation, respect for differences, and community well-being constitutes an important internal foundation for helping and supportive behaviors, constructive expression, information sharing, and public-interest and mutual-aid activities on digital platforms. Further analysis showed that online prosocial self-efficacy and moral responsibility played significant partial mediating roles in the relationship between prosocial civic value orientation and online prosocial participation. This suggests that value orientation is not automatically translated into behavioral practice, but is psychologically transformed through efficacy beliefs and a sense of responsibility. The former strengthens individuals’ beliefs in their ability to help others online and exert a positive influence, whereas the latter enhances their normative awareness of the consequences of online behavior, others’ well-being, and the norms of public interaction. At the same time, public-issue exposure frequency significantly strengthened the positive effect of prosocial civic value orientation on online prosocial participation, indicating that exposure to public-issue information on digital platforms provides an important contextual condition for value activation and behavioral enactment. Overall, college students’ online prosocial participation results from the interaction of value endorsement, psychological mechanisms, and digital contexts. From the perspective of value orientation, psychological transformation, and behavioral enactment, this study extends the explanatory framework of online prosocial behavior research and provides empirical evidence for universities to advance digital citizenship education and online moral education practices.

## Data Availability

The raw data supporting the conclusions of this article will be made available by the authors, without undue reservation.
