# Supplementary material for: From prosocial civic values to online prosocial participation: the roles of self-efficacy, moral responsibility, and public-issue exposure
Source: Front Psychol. 2026 Sep 11;17:1887605. doi: 10.3389/fpsyg.2026.1887605 (PMC13616489; doi:10.3389/fpsyg.2026.1887605)
Supplement: Supplementary file 1 [file Supplementary_file_1.DOC]

**Appendix A. Full Questionnaire Items**

**Instructions**

Dear Participant,

Thank you for taking part in this study. This questionnaire aims to understand college students’ values, perceptions, experiences, and participation in digital environments. There are no right or wrong answers. Please respond to each item based on your actual views and experiences.

Your responses will be used for academic research purposes only. All information will be kept confidential and analyzed anonymously.

**Section A. Prosocial Civic Value Orientation**

**Instructions:** Please indicate the extent to which you agree with each of the following statements.

**Response scale:**
1 = Strongly disagree
2 = Disagree
3 = Neither agree nor disagree
4 = Agree
5 = Strongly agree

| **Item Code** | **Item** |
| --- | --- |
| PCV1 | I believe that, as a member of society, I have a responsibility to pay attention to issues concerning the public interest. |
| PCV2 | I believe that people should support and cooperate with one another when individuals or groups are in need of help. |
| PCV3 | I believe that people from different backgrounds and with different viewpoints should be respected and included in public life. |
| PCV4 | I believe that individual choices should take into account the well-being of the broader community and society. |

**Section B. Online Prosocial Self-Efficacy**

**Instructions:** Please indicate the extent to which you agree with each of the following statements about your abilities in online environments.

**Response scale:**
1 = Strongly disagree
2 = Disagree
3 = Neither agree nor disagree
4 = Agree
5 = Strongly agree

| **Item Code** | **Item** |
| --- | --- |
| OPS1 | I am capable of providing useful support to people who need help through online platforms. |
| OPS2 | Even when disagreements arise, I am capable of engaging in constructive interactions in online environments. |
| OPS3 | I believe that my online expression or actions can have a positive influence on others or the broader online environment. |

**Section C. Moral Responsibility**

**Instructions:** Please indicate the extent to which you agree with each of the following statements.

**Response scale:**
1 = Strongly disagree
2 = Disagree
3 = Neither agree nor disagree
4 = Agree
5 = Strongly agree

| **Item Code** | **Item** |
| --- | --- |
| MR1 | I carefully consider the possible consequences of my online words and actions for other people and the broader online environment. |
| MR2 | When online behavior may affect other people’s well-being, I believe I have a responsibility to act carefully. |
| MR3 | In online public discussions, I believe I have a responsibility to participate in a constructive manner. |
| MR4 | I believe that I should take responsibility for inappropriate online words or actions and for the consequences they may cause. |

**Section D. Public-Issue Exposure Frequency**

**Instructions:** When using social media, campus online communities, or other digital platforms in your daily life, how often do you encounter the following types of content?

**Response scale:**
1 = Never
2 = Rarely
3 = Sometimes
4 = Often
5 = Very frequently

| **Item Code** | **Item** |
| --- | --- |
| PIE1 | Content related to social and public issues. |
| PIE2 | Content related to public-interest initiatives, mutual assistance, requests for help, or volunteer activities. |
| PIE3 | Content related to campus public affairs. |
| PIE4 | Discussions, comments, or exchanges of views concerning public issues. |

**Section E. Online Prosocial Participation**

**Instructions:** When using social media, campus online communities, or other digital platforms in your daily life, how often do you engage in the following behaviors?

**Response scale:**
1 = Never
2 = Rarely
3 = Sometimes
4 = Often
5 = Very frequently

| **Item Code** | **Item** |
| --- | --- |
| OPP1 | Providing information, advice, emotional support, or other forms of help to people in need through online platforms. |
| OPP2 | Expressing opinions in online discussions in a rational, respectful, and constructive manner. |
| OPP3 | Actively sharing useful information or reliable resources that may benefit others. |
| OPP4 | Participating in online mutual-aid activities, public-interest initiatives, or other activities intended to promote collective well-being. |

**Section F. Demographic Information**

Please provide the following background information.

**F1. Gender**

- Male
- Female
- Other / Prefer to self-describe
- Prefer not to say

**F2. Year of Study**

- First year
- Second year
- Third year
- Fourth year

**F3. Place of Origin**

- Urban area
- Rural area

**F4. Only-Child Status**

- Yes
- No
